# 基于复合净化柱-超高效液相色谱-串联质谱法同时测定鱼、虾、蟹中50种兽药残留

**DOI:** 10.3724/SP.J.1123.2024.11017

**Published:** 2025-07-08

**Authors:** Huidan DENG, Xiaofeng JI, Yingping XIAO, Qiang XIA, Hua YANG

**Affiliations:** 1.宁波大学食品科学与工程学院，浙江 宁波 315832; 1. School of Food Science and Engineering，Ningbo University，Ningbo 315832，China; 2.浙江省农业科学院农产品质量安全与营养研究所，浙江 杭州 310021; 2. Institute of Agro-product Safety and Nutrition，Zhejiang Academy of Agricultural Sciences，Hangzhou 310021，China

**Keywords:** 水产品, 兽药残留, 食品安全, 超高效液相色谱-串联质谱法, aquatic products, veterinary drug residues, food safety, ultra-performance liquid chromatography-tandem mass spectrometry （UPLC-MS/MS）

## Abstract

建立了一步式复合净化柱样品前处理方法-超高效液相色谱-串联质谱（UPLC-MS/MS）测定鱼、虾、蟹中喹诺酮类、大环内酯类、硝基咪唑类、磺胺类、酰胺醇类、苯并咪唑类及敌百虫、金刚烷胺、尼卡巴嗪共50种兽药残留的方法。5 g样品经1.0%乙酸乙腈溶液提取，无水硫酸钠盐包去除水分，FAVEX净化柱净化，净化液氮吹至近干，0.2%甲酸水溶液-甲醇（9∶1，v/v）复溶残渣至1.0 mL，过滤膜后待测物经Waters ACQUITY UPLC^®^ BEH C18色谱柱（100 mm×2.1 mm，1.7 μm）分离，在正、负离子模式下以UPLC-MS/MS多反应监测模式采集数据，分别用同位素内标法、基质溶液外标法定量。在最优的仪器参数及前处理条件下进行方法验证，结果表明，目标化合物在各自相应的质量浓度范围内线性关系良好，决定系数（*R* ^2^）均大于0.990 0，检出限（LOD）为0.2～6.0 μg/kg，定量限（LOQ）为0.5～20.0 μg/kg。取鱼、虾、蟹的空白样品进行3个水平的加标回收试验（*n*=6），50种兽药的平均回收率为60.1%～119.7%，相对标准偏差（RSD）为1.11%～15.6%。将该方法应用于150批次实际样品的测定，兽药残留检出率为47.3%，超标率为7.3%，说明水产品中确实存在兽药残留的现象，后续应加强对水产品中兽药残留的风险预警和评估。该方法具有前处理过程简单、检测效率高、有机试剂消耗少、成本低等优点，适用于同时测定鱼、虾、蟹中多种兽药残留，可为水产品风险监测、风险评估和预警提供强有力的技术支持。

## Abstract (en)

China is the world’s largest producer and consumer of aquatic products. Insecticides， as well as veterinary drugs， such as quinolones， macrolides， nitroimidazoles， sulfonamides， and amphenicols， are widely used in aquaculture. These compounds play crucial roles in preventing， controlling， and treating diseases in aquatic organisms， improving feed conversion rates， and promoting the healthy growth of farmed species. However， the illegal use of veterinary drugs， their overuse， and noncompliance with drug withdrawal periods by farmers can lead to aquatic products containing veterinary drug residues. This study focused on fish， shrimp， and crabs with the aim of meeting the green-development needs of China’s aquaculture industry， strengthening risk warnings and preventing veterinary drug residues in aquatic products， enhancing the quality and competitiveness of farmed aquatic products， and promoting the sustainable development of the aquaculture industry. Instrumental working conditions were optimized， and the extraction solvent， extraction method， purification materials， and the redissolution solvent used in the sample-pretreatment process were determined. These efforts led to the establishment of a protocol for the detection of 50 veterinary drug residues， including quinolones， macrolides， nitroimidazoles， sulfonamides， amphenicols， and insecticides， in fish， shrimp， and crabs， which involved one-step pretreatment with a composite purification column in combination with ultra-performance liquid chromatography-tandem mass spectrometry （UPLC-MS/MS）. A 5-g sample was extracted with a 1.0% acetic acid solution， dehydrated with anhydrous sodium sulfate， and purified using a FAVEX purification column， after which the purified solution was nitrogen-blown to near dryness. The residue was re-dissolved in 1.0 mL of 0.2% formic acid aqueous solution-methanol （9∶1， v/v） ， filtered， and then separated using a Waters ACQUITY UPLC^® ^BEH C18 column （100 mm × 2.1 mm， 1.7 μm）. Data were collected in positive- and negative-ion multiple reaction monitoring modes， and quantified using isotopic internal and external standards. All target compounds exhibited good linear relationships within their respective concentration ranges， with coefficients of determination （*R*²） greater than 0.990 0. Average recovery rates of 60.1%–117.8% were determined for the 50 veterinary drugs in the fish matrix， with RSDs of between 1.89% and 15.0%， while the shrimp matrix delivered average recovery rates of 60.2%–119.7%， with RSDs of 1.11%–15.6%， and the crab matrix exhibited average values of 60.7%–119.7% and 2.94%–15.0%， respectively. The developed method is advantageous owing to its simple pretreatment step， high throughput， low organic-solvent consumption， good accuracy， high sensitivity， and cost-effectiveness. One hundred and fifty batches of aquatic products， including 50 batches of fish， 50 batches of shrimp， and 50 batches of crab， were risk-screened for 50 veterinary drug residues using the established detection method. Veterinary drug residues were detected at a rate of 47.3%， with an over-standard rate of 7.3%. Specifically， the fish samples exhibited a detection rate of 66.0%， with contamination concentrations of 1.20–257.1 μg/kg. In contrast， the shrimp samples exhibited a detection rate of 18.0%， with contamination concentrations of 1.10–8.60 μg/kg， while values of 22.0% and 3.00–7.30 μg/kg， respectively， were determined for the crab samples. This study identified a total of seven veterinary drug residues， namely pefloxacin， norfloxacin， ciprofloxacin， enrofloxacin， sarafloxacin， sulfadimethoxine， and sulfamethoxazole， with detection rates of 0.67%， 5.3%， 12.7%， 20.7%， 4.6%， 2.00%， and 1.33%， respectively. The detection method established in this study provides strong technical support for monitoring and assessing aquatic-product risks in a timely manner， and is expected play an important role in ensuring food safety， protecting consumer health， and promoting the healthy development of the aquatic-product industry.

水产品因富含优质蛋白及生物活性物质，已成为人类膳食结构的重要组成部分。据统计，2023年我国水产品交易总额近千亿元，同比增长5%^［1］^，稳居全球水产养殖与消费首位。为保障水产养殖效益，喹诺酮类、大环内酯类等兽药被广泛应用于疾病防控与生长调节^［2］^。然而，兽药在生物体内的吸收、分布、代谢及排泄过程具有复杂性，若养殖环节存在违规超量使用或忽视休药期等行为，将导致水产品中药物残留超标。此类残留物不仅可通过食物链在人体内蓄积，诱发肠道菌群紊乱、耐药菌株增殖及潜在“三致”风险^［3，4］^，其代谢产物还会经环境介质迁移转化，加剧水体与土壤污染^［5］^，形成“养殖-环境-健康”连锁风险。

为规范水产品质量安全管理，不同国家和组织均发布了食品中兽药残留限量标准。国际食品法典委员会（CAC）发布了兽药最大残留限量标准CX/MRL 2-2021《食品中兽药残留最大残留限量和风险管理建议》^［6］^，欧盟有较CAC更严格的食品安全法规，2009年发布的No 37/2010《食品中药物最大残留限量》^［7］^至今已经过多次修订，涉及多种药物的最大残留限量。我国目前的食品相关标准体系主要涵盖了通用要求、产品规范、检测方法等多个方面；随着我国食品安全国家标准的发布，食品中兽药残留检测方法在检测手段^［8］^、样品种类和检测药物种类较以往有了明显突破^［9］^。科学评估并明确规定兽药在食品中的最大允许残留量，是保障消费者健康和促进国际贸易的必要措施^［10］^。《食品安全国家标准 食品中兽药最大残留限量》（GB 31650-2019）^［11］^、《食品安全国家标准 食品中41种兽药最大残留限量》（GB 31650.1-2022）^［12］^等基本覆盖了我国常用的兽药种类和居民日常可食性动物组织。

水产品检测对于保障食品安全、维护消费者权益、促进水产品行业健康发展具有重要意义。目前，食品中兽药残留检测前处理方法有液液萃取法、固相萃取法、基质分散萃取法、QuEChERS方法^［13］^等，兽药残留检测技术有酶联免疫法、生物法、气相色谱法、高效液相色谱法、高效液相色谱-串联质谱法^［14，15］^等。生物法和酶联免疫法^［16，17］^操作简单快速，但生物法的材料选择具有一定的局限性，免疫法的检测结果与抗体来源密切相关，样品基质中细胞因子可溶性受体可能影响特异性抗体的结合。高效液相色谱技术适用于极性、热不稳定或大分子化合物检测^［18］^，可检测痕量物质，满足国家食品安全标准的要求，但分析时间较长，传统高效液相色谱每个样本需要10~30 min左右，超高效液相色谱可缩短分析时间，但需更高柱压和更小粒径的色谱柱。气相色谱法^［19］^成本低，但只适合可气化或衍生的小分子药物，高效液相色谱-串联质谱法^［20］^可以对兽药残留进行定性和定量分析，复杂基质的样品分离效果也很好，可同时检测食品中多种兽药残留，是目前兽药残留检测最受欢迎的方法之一。然而，大部分国家检测标准多以某一类药为主建立前处理方法，无法满足大批量水产品中多种兽药残留筛查分析的要求，同时也无法发挥HPLC-MS/MS的优势。为满足我国水产养殖业绿色发展的需求，强化水产品中兽药残留风险预警和防控，提升水产品品质和市场竞争力，推动水产行业可持续发展，建立一种简单、高效、环保的水产品中多种兽药残留检测方法成为一种必然趋势。

本研究通过系统优化色谱与质谱条件，结合一步式复合净化柱前处理技术，利用超高效液相色谱-串联质谱法（UPLC-MS/MS）建立了鱼、虾、蟹中50种兽药残留的高通量同步检测方法。该方法显著提升了多残留分析的效率与准确性，为水产品中兽药残留的精准风险监测、科学评估与实时预警提供了高效、快速、便捷且环保的技术手段，对保障食品安全和推动绿色检测技术发展具有重要意义。

## 1 实验部分

### 1.1 仪器、试剂与材料

AB SCIEX QTRAP 5500三重四极杆液相色谱-质谱联用仪（美国AB SCIEX公司）；KQ-250B 型超声波清洗器（中国昆山市超声仪器有限公司）；VX-Ⅲ多管涡旋振荡器（中国北京安简科技有限公司）；AUTO EVA全自动氮吹仪（中国厦门睿科集团股份有限公司）；BIOFUGE PRIMO R型高速离心机（美国Thermo Fisher Scientific公司）。

50种兽药标准溶液：16种喹诺酮类混合标准溶液、5种大环内酯类混合标准溶液、5种硝基咪唑类混合标准溶液、15种磺胺类混合标准溶液、3种酰胺醇类混合标准溶液、3种苯并咪唑类混合标准溶液和敌百虫、金刚烷胺、尼卡巴嗪3种单标溶液。所有兽药质量浓度均为100 μg/mL，其中敌百虫、尼卡巴嗪溶剂为乙腈，其余兽药溶剂均为甲醇（天津阿尔塔科技有限公司）。

9种兽药内标溶液：3种喹诺酮类混合标准溶液（诺氟沙星-d5、环丙沙星-d8、恩诺沙星-d5）、2种磺胺类混合标准溶液（磺胺邻二甲氧嘧啶-d3、磺胺间二甲氧基嘧啶-d6）、1种金刚烷胺-d15和3种酰胺醇类混合标准（氯霉素-d5、氟苯尼考-d3、甲砜霉素-d3）。所有兽药内标质量浓度均为100 μg/mL，溶剂均为甲醇（天津阿尔塔科技有限公司）。

甲醇和乙腈（色谱纯，美国Merck公司）；甲酸（色谱纯）、乙酸（色谱纯）和无水硫酸钠（纯度≥99.0%）（上海凌峰化学试剂有限公司）；甲酸铵（纯度≥99.0%，比利时ACROS公司）。

FAVEX-NM-Aqu50净化柱（5 mL/5 g）（上海巨研科技股份有限公司）； Milli-Q A10 超纯水系统（美国 Millipore 公司）；0.22 μm有机膜（13 mm，北京迪马科技有限公司）。

鱼、虾、蟹样品随机抽取自浙江省水产流通市场。样品采集后迅速置于保温箱，保持0～10 ℃的低温状态送至实验室，整个过程不超过12 h。取水产品可食部分用高速组织搅拌机均质备用，一份用于检验，另一份作为留样，每份样品200 g。

### 1.2 标准溶液的配制

标准工作溶液：准确吸取质量浓度均为 100 μg/mL的各标准溶液适量，用甲醇稀释分别配成1 μg/mL的标准工作溶液，避光4 ℃冷藏保存，有效期1个月。

内标标准工作溶液：准确吸取质量浓度均为 100 μg/mL的各氘代同位素内标标准溶液适量，用甲醇稀释分别配成1 μg/mL内标标准工作液，避光4 ℃冷藏保存，有效期1个月。

溶剂系列工作溶液：准确量取适量标准工作溶液和内标标准工作溶液，用0.2%甲酸水溶液-甲醇（9∶1，v/v）稀释成质量浓度为1、2、5、10、20、50、100 μg/L的溶剂系列工作溶液，内标浓度均为10 μg/L，供UPLC-MS/MS测定，制作溶剂标准曲线。

基质系列工作溶液：准确量取适量标准工作溶液，用鱼、虾、蟹的空白基质液分别稀释成质量浓度为1、2、5、10、20、50、100 μg/L的基质标准系列工作溶液，供UPLC-MS/MS测定，根据基质类别，制作基质标准曲线。

### 1.3 实验方法

#### 1.3.1 样品前处理

精确称取均质后的样品5.00 g（精确至0.01 g）于50 mL离心管中，准确加入10 mL 1%（v/v）乙酸乙腈，振荡5 min，超声10 min，加入3 g无水硫酸钠，振荡15 s，8 500 r/min离心5 min，吸取4 mL上清液，过FAVEX-NM-Aqu50净化柱，准确移取2 mL滤液于10 mL玻璃管中，于40 ℃下用氮气吹至近干。准确加入1 mL 0.2%甲酸水溶液-甲醇（9∶1，v/v）复溶，涡旋混匀30 s，用0.22 μm有机滤膜过滤，UPLC-MS/MS上机分析。

#### 1.3.2 仪器条件

色谱条件 ACQUITY UPLC^®^ BEH C18色谱柱（100 mm × 2.1 mm，1.7 μm；美国Waters公司）；流动相：A为甲醇，B为2 mmol/L甲酸铵水溶液（含0.1%甲酸）；梯度洗脱程序：0～3.0 min，95%B～80%B；3.0～7.5 min，80%B～1%B；7.5～10.0 min，1%B；10.0～10.1 min，1%B～95%B；10.1～12.0 min，95%B。进样体积为5.0 μL；流速为0.4 mL/min；柱温为40 ℃。

质谱条件 离子源为电喷雾离子源，正离子模式（ESI^+^）和负离子模式（ESI^-^）；质谱扫描方式：多反应监测（multiple reaction monitoring，MRM）；离子源喷雾电压为+5 500 V/-4 500 V，离子源温度为500 °C，气帘气压力为276 kPa（40 psi）；雾化气压力为379 kPa（55 psi）；辅助气压力为345 kPa（50 psi）。离子源气体为空气，碰撞气体为高纯度氮气（纯度99.99%）。50种兽药的质谱参数详见表1。

**表1:** 50种目标化合物及9种内标化合物的质谱参数

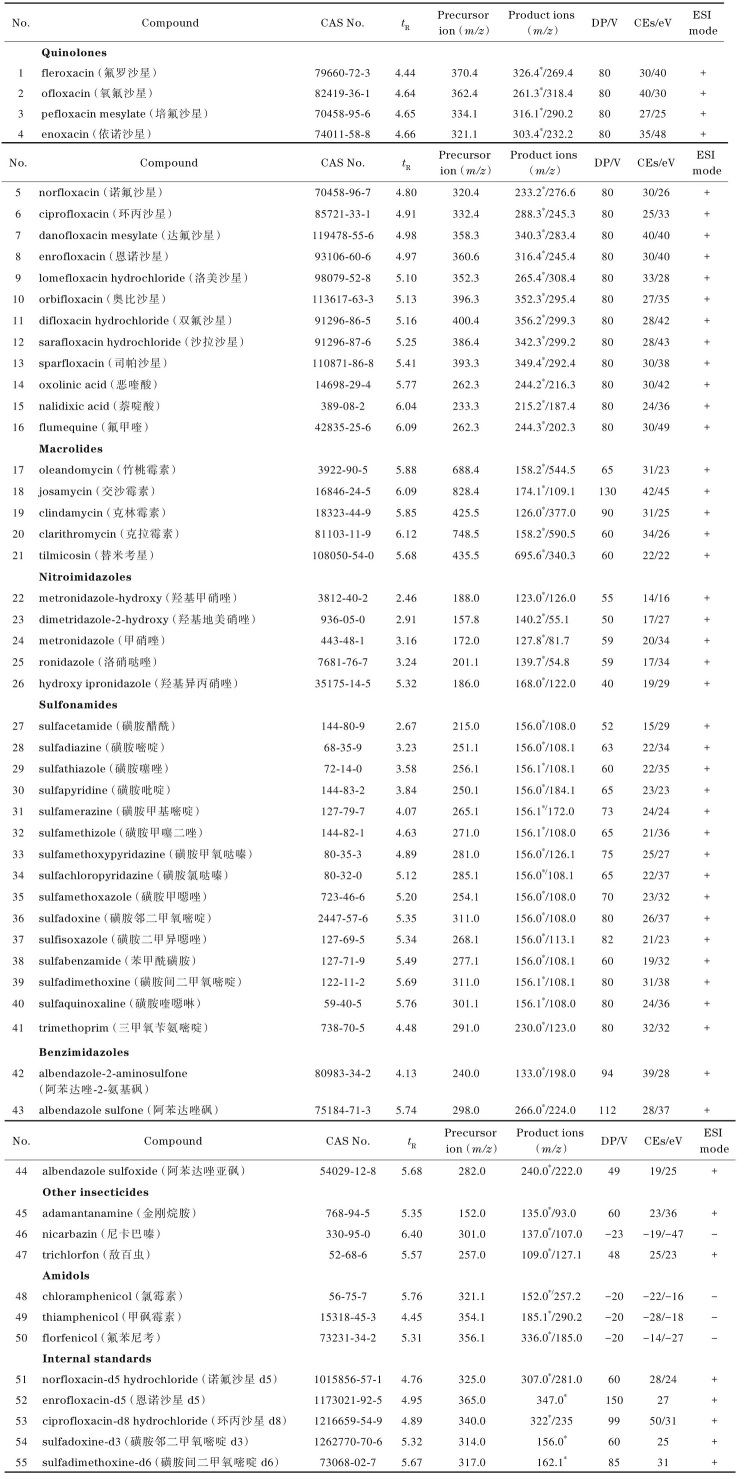

* Quantitative ion； DP： decluster potential； CEs： collision energies.

## 2 结果与讨论

### 2.1 LC-MS条件优化及基质效应

#### 2.1.1 色谱条件的优化

测试了5种流动相体系，分别为2 mmol/L甲酸铵水溶液（含0.1%甲酸）-甲醇、5 mmol/L甲酸铵水溶液（含0.1%甲酸）-甲醇、5 mmol/L乙酸铵水溶液（含0.1%甲酸）-甲醇、0.1%甲酸水溶液-甲醇和0.1%甲酸水溶液-乙腈。研究结果表明，当乙腈作为流动相时，相比甲醇，酰胺醇类兽药响应较低、环丙沙星有拖尾现象，因此，有机相选择甲醇。正离子模式下，0.1%甲酸水溶液作为流动相时，可促进目标化合物离子化。尼卡巴嗪和酰胺醇类兽药为负离子模式，甲酸会抑制其离子化效率。为达到正负离子模式同时检测多种兽药残留的结果，在甲酸水溶液中加入一定的甲酸铵或乙酸铵。研究结果表明，水相为2 mmol/L甲酸铵水溶液（含0.1%甲酸）时，绝大多数兽药具有良好的峰形和响应强度。因此，流动相体系选择2 mmol/L甲酸铵水溶液（含0.1%甲酸）-甲醇。

#### 2.1.2 质谱条件的优化

在ESI^+^或ESI^-^模式下，依次针泵进样50种1.0 μg/mL的兽药标准溶液。先采用质谱仪一级质谱扫描，以确定目标化合物的母离子及质荷比（*m/z*）。然后将扫描模式更改为二级质谱扫描，通过调节碰撞电压，选择信号响应最强的子离子作为定量离子，选择信号最稳定的子离子作为定性离子，最后在MRM模式下优化去簇电压和碰撞能量，进而达到最佳的检测灵敏度。

#### 2.1.3 基质效应

基质效应（ME）由共萃取物对目标化合物的电离作用引起，影响分析方法的准确性和灵敏度^［21］^。常用的评价基质效应的公式为ME=（基质标准曲线斜率/溶剂标准曲线斜率-1）×100%，当|ME|≤20%时表现为弱基质效应，20%<|ME|≤50%时表现为中等基质效应，|ME|>50% 时表现为强基质效应^［22，23］^。

经仪器测定，将溶剂系列工作溶液和基质系列工作溶液中各化合物的峰面积（*y*）与其质量浓度（*x*）进行线性拟合。计算出基质效应如图1所示，鱼肉基质中培氟沙星、磺胺噻唑、甲砜霉素和氟苯尼考ME均大于50%，表明基质对这些兽药具有强烈的增强效应；虾肉基质中氯霉素的ME为154.9%，基质对氯霉素具有增强效应；蟹肉基质中有78.0%的兽药ME都在-20%以下，存在基质抑制现象。因此，本实验分别采用内标法和基质溶液外标法定量分析样品中的50种兽药，从而消除基质效应对目标化合物定量分析的影响。

**图1:**
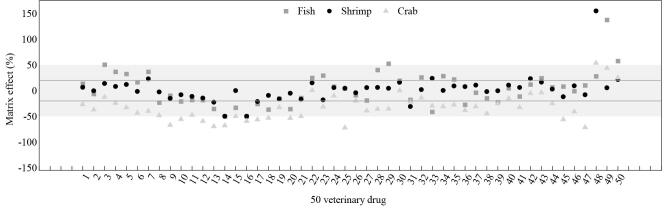
鱼肉、虾肉和蟹肉中50种兽药的基质效应

### 2.2 前处理条件优化

本研究以6类兽药和敌百虫、金刚烷胺、尼卡巴嗪的平均回收率（*n*=6）为指标，空白鳕鱼肉为基质样品，在5 μg/kg的添加水平下优化了提取溶剂、提取方式、净化方式和复溶剂。

#### 2.2.1 提取溶剂

样品中兽药残留测定中提取溶剂的选择至关重要。兽药残留检测中常用的提取溶剂有甲醇、乙腈和乙酸乙酯等。本研究分别考察了甲醇、乙腈、0.5%乙酸乙腈、1%乙酸乙腈、1.5%乙酸乙腈的提取效果。实验结果表明，甲醇作提取溶剂时，提取液中杂质较多，干扰目标化合物的出峰效果，6类兽药平均回收率偏低，有38.0%的兽药回收率在70%以下。乙腈对多数兽药具有良好的溶解性和较高的提取率，而且利于沉降蛋白质，同时可避免从水产品组织中提取较多的脂肪^［24，25］^。本研究中，采用乙腈为提取溶剂时，50种兽药中有52.0%的兽药平均回收率为70%～120%。根据前期文献，当乙腈中加入酸性溶剂时，不但可以破坏细胞组织的结构，使目标分析物能够很好地分离出来，还可以改变样品体系的pH，从而改变兽药在水相-有机相之间的分配比，增强兽药被乙腈层提取的效率^［26］^。本研究结果表明，以1%乙酸乙腈作为提取剂时，50种兽药的回收率普遍较高，符合回收率要求的兽药占68.0%，高于0.5%乙酸乙腈（62.0%）和1.5%乙酸乙腈（66.0%）的提取回收率。其中，当酸性溶剂的含量增加到1%时，诺氟沙星、环丙沙星、氟苯尼考的回收率明显提高。因此，选择1%乙酸乙腈作为提取剂。不同提取溶剂对50种兽药回收率及标准偏差的影响见附图S1（www.chrom-China.com）。

#### 2.2.2 提取方式

在兽药残留分析的前处理阶段，核心要求在于达成目标化合物与样品基质的高效分离，其中提取方法的选择及其优化是影响目标物萃取效率的关键环节。水产品基质成分复杂，质地黏稠、不易散开，严重影响待测化合物的提取效率。本实验采用多管涡旋振荡器和超声波清洗器辅助设备，分别考察了5种提取方式，分别为（A）振摇1 min，不超声；（B）振摇5 min，不超声；（C）振摇10 min，不超声；（D）振摇5 min，超声10 min；（E）振摇5 min，超声20 min。

对比了3种振摇时间（1、5、10 min）对样品基质分散状态的影响，实验结果表明，样品基质单独振摇1 min时，样品基质部分散开，仍存在凝结成团现象，振摇5 min和10 min时，样品基质可均匀地分散在提取液中。通过对比50种兽药的平均加标回收率可以看出，振摇1 min时，磺胺甲基嘧啶、三甲氧苄氨嘧啶、洛美沙星和恩诺沙星的回收率仅为54.4%～69.2%，振摇5 min时，上述兽药回收率提高至65.2%～114.1%，74.0%的兽药平均回收率在70%～120%，优于单独振摇1 min（72.0%）和10 min（66.0%），72.0%的兽药回收率重复性（RSD）小于20.0%，优于单独振摇1 min的重复性（58.0%）。基于样品基质分散程度和回收率结果，本研究最终选择振摇时间为5 min。

超声处理方式可以提高化合物的传质速率，缩短提取时间，在食品检测领域有广泛的应用^［27］^。对比了不同超声时间（10、20 min）对目标化合物回收率的影响，实验结果表明，振摇5 min，超声10 min，86.0%的兽药回收率为70%～120%。相对于单独振摇5 min提取，加入超声处理10 min后，羟基甲硝唑的回收率从53.3%提高至60.8%，洛硝哒唑的回收率从57.6%提高至77.4%，大环内酯类兽药回收率提高至87.0%～125.2%。但当超声时间延长至20 min时，敌百虫、阿苯达唑亚砜、金刚烷胺和尼卡巴嗪的平均回收率低于70%，存在明显的基质抑制现象；克拉霉素、竹桃霉素、氯霉素的回收率为121.2%～149.2%，存在明显的基质增强现象。综合考虑目标分析物的回收率、实际操作等因素，本研究采用振摇5 min，超声10 min的提取组合。不同振摇和超声时间对50种兽药回收率和RSD的影响见附图S2。

#### 2.2.3 净化方式

水产品基质复杂，含有丰富的蛋白质、固醇类和脂肪等干扰物质，不仅影响药物的分析定量，而且会缩短色谱柱的使用寿命，增加仪器维护成本^［28］^。本研究比较了Oasis HLB柱（6 mL/200 mg）、Oasis PRIME HLB柱（6 mL/200 mg）、FAVEX-NM-Aqu50柱和PEP-2柱（6 mL/200 mg）等净化柱对鳕鱼肉中多种目标化合物的净化效果，不同净化材料对50种兽药回收率和标准偏差的影响见附图S3。实验结果表明，Oasis HLB柱对样品中部分喹诺酮类、磺胺类和酰胺醇类兽药具有良好的净化效果，但无法保留敌百虫、硝基咪唑类和苯并咪唑类等兽药，回收率仅为1.5%～42.3%。Oasis PRIME HLB、FAVEX-NM-Aqu50和PEP-2柱均为过滤型去除杂质净化柱，无需活化、淋洗和洗脱等步骤。综合比较3种净化柱，PEP-2柱和FAVEX-NM-Aqu50柱净化效果相当，但PEP-2柱对敌百虫和苯并咪唑类兽药保留效果较差，如敌百虫和阿苯达唑砜，回收率仅为25.5%和19.4%。使用PEP-2柱过滤的净化液经氮吹近干后复溶，复溶液较另外两组复溶液浑浊，脂质等干扰物去除效果差。基于样品基质净化效果和加标回收率的影响研究，本研究最终选择FAVEX-NM-Aqu50净化柱作为净化材料，76.0%的兽药平均回收率为70%～120%。

#### 2.2.4 复溶剂

复溶剂会影响色谱分离效果和质谱响应，根据目标化合物的性质和提取后的基质成分，可以选择不同的复溶剂^［29，30］^。考察了甲醇-水（1∶1，v/v，含0.1%甲酸）、0.2%甲酸水溶液-甲醇（9∶1，v/v）和甲醇-水（1∶1，v/v）3种复溶剂的效果。实验结果表明，3种复溶剂下的目标化合物峰形没有明显差异，采用3种复溶剂的峰面积没有明显差异（*P*>0.05）。从加标回收率结果分析，0.2%甲酸水溶液-甲醇（9∶1，v/v）做复溶剂时，82.0%的目标化合物加标回收率为70%～120%，优于甲醇-水（1∶1，v/v）含0.1%甲酸（64.0%）和甲醇-水（1∶1，v/v）（60.0%），88.0%的兽药RSD小于20.0%，优于甲醇-水（1∶1，v/v，含0.1%甲酸）（80.0%）和甲醇-水（1∶1，v/v）（82.0%）。不同复溶剂对50种兽药回收率和RSD的影响结果如图2所示。本研究选择0.2%甲酸水溶液-甲醇（9∶1，v/v）作为复溶剂。

**图2:**
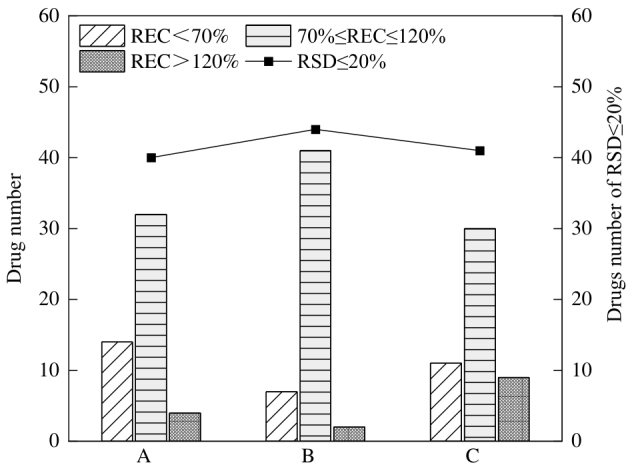
不同复溶剂对50种兽药（5 μg/kg）回收率和RSD的影响

### 2.3 方法学评估

#### 2.3.1 线性关系、检出限和定量限

针对采用基质外标法定量的15种兽药（见表2），采用1.3.1节前处理方法得到鱼、虾、蟹的空白基质液，按照1.2节方法配制基质标准溶液，15种兽药的质量浓度为横坐标（*x*），以15种兽药对应的定量离子峰面积为纵坐标（*y*）；针对内标法定量的35种兽药（见表2），按照1.2节方法配制溶剂标准溶液，35种兽药的质量浓度为横坐标（*x*），35种兽药与对应内标的峰面积之比为纵坐标（*y*）。实验结果表明，喹诺酮类、大环内酯类和磺胺类兽药的线性范围为2～100 μg/kg，其中磺胺噻唑和磺胺甲基嘧啶的线性范围为5～100 μg/kg。硝基咪唑类兽药线性范围为1～100 μg/kg，其中羟基甲硝唑的线性范围为20～100 μg/kg；羟基地美硝唑的线性范围为10～100 μg/kg；羟基异丙硝唑的线性范围为5～100 μg/kg。敌百虫、尼卡巴嗪和酰胺醇类兽药的线性范围为1～100 μg/kg，阿苯达唑亚砜和阿苯达唑-2-氨基砜的线性范围为2～100 μg/kg；阿苯达唑砜的线性范围为10～100 μg/kg；金刚烷胺的线性范围为5～100 μg/kg。本研究中，检测分析的各个目标化合物在相应质量浓度范围内线性关系良好，决定系数（*R* ^2^）均大于0.990 0。

**表2:** 50种兽药在鱼、虾、蟹基质中的检出限、定量限、线性范围、决定系数及对应的内标物

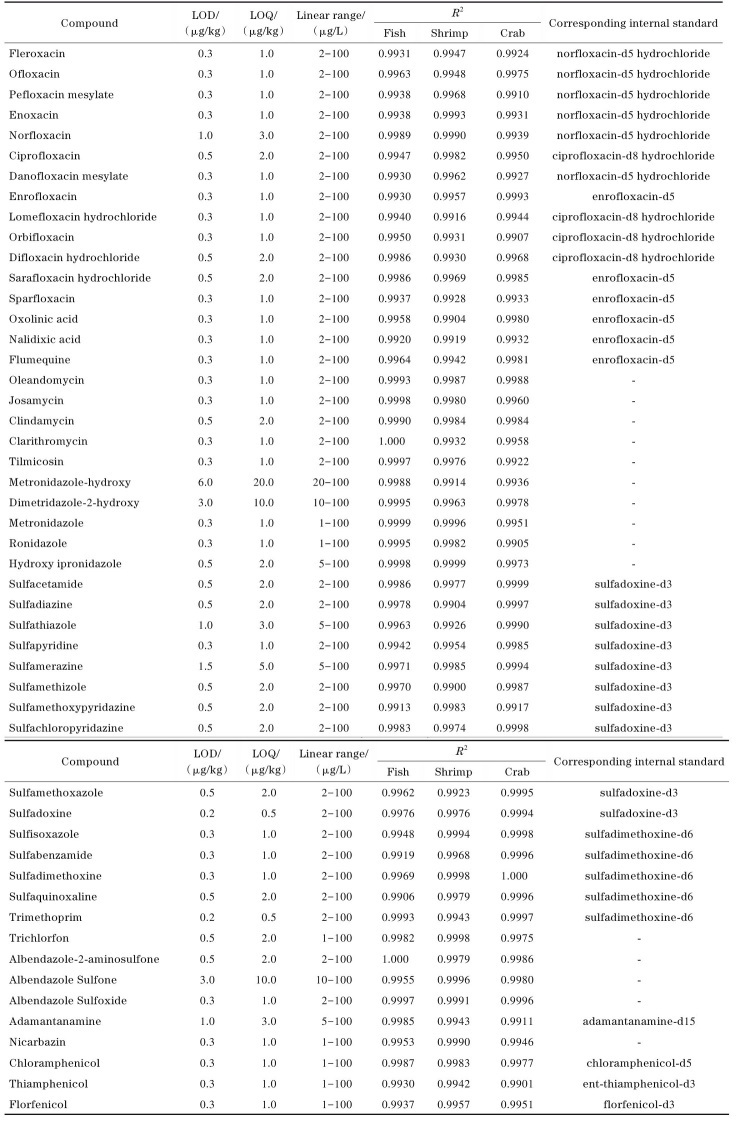

-： quantified by external standard method.

不同样品的基质效应有所不同，本研究选用鱼样品基质来确定本方法的检出限和定量限。本研究以信噪比为3（*S/N*≥3）确定方法的检出限（LOD），以信噪比为10（*S/N*≥10）确定方法的定量限（LOQ）。实验结果表明，喹诺酮类、磺胺类和大环内酯类兽药的检出限为0.2～1.5 μg/kg，定量限为0.5～5.0 μg/kg，硝基咪唑类、苯并咪唑类、酰胺醇类及其余兽药的检出限为0.3～6.0 μg/kg，定量限为1.0～20.0 μg/kg。50种兽药在鱼、虾、蟹基质中的检出限、定量限、线性范围、相关系数及对应的内标物见表2，其中未标记内标的兽药采用基质外标法定量。

#### 2.3.2 回收率和精密度

分别取鳕鱼、南美白对虾和青蟹的空白样品，按照1.3.1节前处理步骤做3个水平的加标回收试验，每个水平做6个平行，计算各待测兽药的平均回收率和RSD，结果见附表S1。实验结果表明，50种兽药在鱼肉基质中的平均回收率为60.1%～117.8%，RSD为1.89%～15.0%；在虾肉基质中的平均回收率为60.2%～119.7%，RSD为1.11%～15.6%；在蟹肉基质中的平均回收率为60.7%～119.7%，RSD为2.94%～15.0%。本研究所建立方法的准确性和精密度能够达到兽药多残留检测的定性、定量要求，符合GB/T 27417-2017《合格评定 化学分析方法确认和验证指南》^［31］^中对回收率和精密度的要求。

### 2.4 方法对比

现行国家标准对水产品中不同类别兽药残留的测定有多个独立方法，要完成水产品中上述50种兽药残留的测定，至少需用到《中华人民共和国农业部公告第 1077号》^［32］^、《食品安全国家标准 水产品中大环内酯类药物残留量的测定 液相色谱-串联质谱法》（GB 31660.1-2019）^［33］^、《食品安全国家标准 动物性食品中硝基咪唑类药物残留量的测定 液相色谱-串联质谱法》（GB 31658.23-2022）^［34］^、《食品安全国家标准 动物性食品中酰胺醇类药物及其代谢物残留量的测定 液相色谱-串联质谱法》（GB 31658.20-2022）^［35］^等国家标准方法，本研究建立的方法展现出显著的技术集成优势。此外，通过与文献报道的检测方法进行比较（表3），本研究建立的方法只需一步提取和净化，操作简单，成本相对较低，且有机溶剂消耗量少，对环境更加友好，可满足实际检测需要。

**表3:** 本研究建立的方法与文献中水产品中兽药残留检测方法的对比

Sample type	Number of drugs	Extraction method	Purification	Organic solvent volume/mL	Ref.
Shrimp	52	extract with acetonitrile （1% formic acid） for 30 min， 1 time	QuEChERS EMR-Lipid dSPE， 1 time	14	［^36^］
Fish and shrimp	27	extract with acetonitrile （0.1% formic acid） for 20 min， 2 times	QuEChERS， 3 times	10	［^37^］
Fish	15	extract with acetonitrile for 30 min， 2 times	LLE， 2 times	48	［^38^］
Fish， shrimp， and crab	50	extract with acetonitrile （1% acetic acid） for 15 min， 1 time	SPE， 1 time	10	this work

### 2.5 实际样品测定

采用本研究建立的检测方法对150批次的水产品中50种兽药残留开展风险筛查分析，样品类型包括50批次鱼类（鲫鱼、鳊鱼、大黄鱼）、50批次虾类（哈氏仿对虾、南美白对虾）和50批次蟹类样品（青蟹、梭子蟹）。结果表明，在150批次水产品中，兽药残留检出率为47.3%，超标率为7.3%。其中，鱼类样品中兽药残留检出率为66.0%，污染水平为1.20～257.1 μg/kg；虾类样品中兽药残留检出率为18.0%，污染水平为1.10～8.60 μg/kg；蟹类样品中兽药残留检出率为22.0%，污染水平为3.00～7.30 μg/kg。本研究中，共有7种兽药残留检出，分别为培氟沙星、诺氟沙星、环丙沙星、恩诺沙星、沙拉沙星、磺胺间二甲氧嘧啶和磺胺甲基嘧啶，检出率分别为0.67%、5.3%、12.7%、20.7%、4.6%、2.00%和1.33%。

根据GB 31650-2019的规定，鱼肉中恩诺沙星（残留标志物为恩诺沙星和环丙沙星）的最大残留限量为100 μg/kg，磺胺类药物的最大残留限量为100 μg/kg。实际样品筛查分析发现，鱼类样品中检出恩诺沙星、环丙沙星、磺胺间二甲氧嘧啶和磺胺甲基嘧啶残留，检出水平为1.20～257.1 μg/kg，其中有2批次鳊鱼样品检出磺胺甲基嘧啶超标，检出水平分别为107.7 μg/kg和257.1 μg/kg；虾类样品中检出恩诺沙星和沙拉沙星残留检出水平为1.10～8.60 μg/kg；蟹类样品中有2批次样品检出恩诺沙星，检出水平分别为6.70 μg/kg和7.30 μg/kg，1批次梭子蟹检出培氟沙星，检出水平为4.90 μg/kg，18批次青蟹中33.3%的样品检出诺氟沙星，检出水平为3.00～7.10 μg/kg。鱼、虾和蟹样品中兽药残留污染情况参见图3。

**图3:**
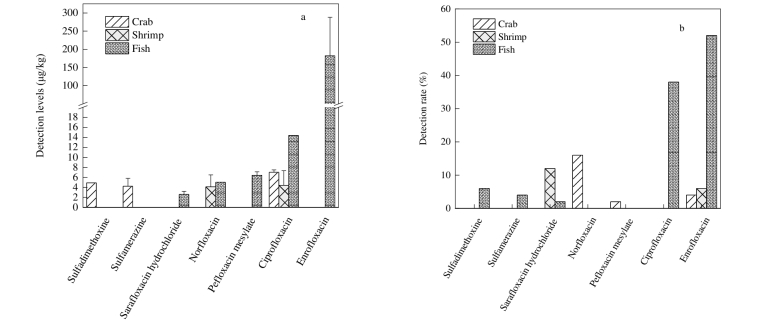
鱼、虾和蟹样品中的兽药残留（a）平均检出水平和（b）检出率 （*n*=3）

## 3 结论

本研究建立了一步式复合净化柱前处理方法结合超高效液相色谱-串联质谱法测定鱼、虾和蟹中喹诺酮类、大环内酯类、硝基咪唑类、磺胺类、酰胺醇类和、苯并咪唑类及敌百虫、金刚烷胺、尼卡巴嗪共50种兽药残留的检测方法，该方法具有前处理操作简便、高通量、有机试剂消耗少，准确性好、灵敏度高和成本低等优点，可为水产品风险监测、风险评估和预警提供强有力的技术支持，同时将在保障食品安全、维护消费者健康、助力水产品产业健康发展等方面发挥重要作用。
